# Asymptomatic or mild symptomatic SARS-CoV-2 infection elicits durable neutralizing antibody responses in children and adolescents

**DOI:** 10.1172/jci.insight.150909

**Published:** 2021-09-08

**Authors:** Carolina Garrido, Jillian H. Hurst, Cynthia G. Lorang, Jhoanna N. Aquino, Javier Rodriguez, Trevor S. Pfeiffer, Tulika Singh, Eleanor C. Semmes, Debra J. Lugo, Alexandre T. Rotta, Nicholas A. Turner, Thomas W. Burke, Micah T. McClain, Elizabeth A. Petzold, Sallie R. Permar, M. Anthony Moody, Christopher W. Woods, Matthew S. Kelly, Genevieve G. Fouda

**Affiliations:** 1 Duke Human Vaccine Institute,; 2 Department of Pediatrics, Division of Infectious Diseases,; 3 Children’s Health & Discovery Institute, Department of Pediatrics, and; 4 Children’s Clinical Research Unit, Department of Pediatrics, Duke University School of Medicine, Durham, North Carolina, USA.; 5 Medical Scientist Training Program, Department of Molecular Genetics and Microbiology, Duke University, Durham, North Carolina, USA.; 6 Department of Pediatrics, Division of Pediatric Critical Care Medicine, and; 7 Department of Medicine, Division of Infectious Diseases, Duke University School of Medicine, Durham, North Carolina, USA.; 9 Durham Veterans Affairs Medical Center, Durham, North Carolina, USA.; 8 Center for Applied Genomics and Precision Medicine, Duke University, Durham, North Carolina, USA.; 10 Department of Pediatrics, Weill Cornell School of Medicine, New York City, New York, USA.

**Keywords:** Immunology, Infectious disease, Adaptive immunity

## Abstract

As SARS-CoV-2 continues to spread globally, questions have emerged regarding the strength and durability of immune responses in specific populations. In this study, we evaluated humoral immune responses in 69 children and adolescents with asymptomatic or mild symptomatic SARS-CoV-2 infection. We detected robust IgM, IgG, and IgA antibody responses to a broad array of SARS-CoV-2 antigens at the time of acute infection and 2 and 4 months after acute infection in all participants. Notably, these antibody responses were associated with virus-neutralizing activity that was still detectable 4 months after acute infection in 94% of children. Moreover, antibody responses and neutralizing activity in sera from children and adolescents were comparable or superior to those observed in sera from 24 adults with mild symptomatic infection. Taken together, these findings indicate that children and adolescents with mild or asymptomatic SARS-CoV-2 infection generate robust and durable humoral immune responses that can likely contribute to protection from reinfection.

## Introduction

Severe acute respiratory syndrome coronavirus 2 (SARS-CoV-2), the etiological agent of coronavirus disease 2019 (COVID-19), has caused more than 180 million infections and nearly 3.8 million deaths globally (1). The effectiveness and durability of the immune responses induced by SARS-CoV-2 infection has major implications for the risk of reinfection and the establishment of herd immunity. Studies conducted among adult populations suggest that there is substantial variability in SARS-CoV-2 humoral immune responses based on patient factors and characteristics of the initial illness. In a study of more than 30,000 individuals in New York City who tested positive for SARS-CoV-2 infection by PCR, more than 90% had detectable IgG antibodies against the viral spike protein within 30 days of SARS CoV-2 acute infection, irrespective of the severity of the initial illness (2). More recent studies have shown that antibody responses in adults are durable, with reports of detectable SARS-CoV-2–specific antibodies up to 6–8 months after acute infection (3). Several prior studies reported an association between the durability of antibody responses and the severity of the acute infection. For example, Long and colleagues found that 40% of the asymptomatic adults and 13% of the symptomatic adults with initial antibody responses seroreverted within 2 months after acute infection (4). Conversely, Kong and colleagues reported that individuals with mild or moderate symptoms had earlier and more robust antibody responses than individuals with severe illness during the 40 days after symptom onset (5). Taken together, these studies demonstrate the marked variability of immune responses within different populations and highlight a potential association between illness severity and the development of immune responses to SARS-CoV-2.

Epidemiological data from around the world indicate that children and adolescents with SARS-CoV-2 infection typically have milder illness courses than adults (6–8). Notably, SARS-CoV-2 infection in children and adolescents is often asymptomatic or associated with such minor symptoms that children infrequently come to medical attention (9, 10). The varied clinical manifestations of SARS-CoV-2 infection among children and adults suggest that age may modify the host response to SARS-CoV-2, as has previously been demonstrated for several other viruses (11–17). To date, studies of SARS-CoV-2 immune responses in pediatric populations have focused primarily on children hospitalized for severe COVID-19 or who developed multisystem inflammatory syndrome in children (MIS-C), a potentially life-threatening inflammatory condition that can occur after SARS-CoV-2 infection (18–22). While these studies provide important insights into the immune responses of children and adolescents who develop these rare manifestations of SARS-CoV-2 infection, surprisingly little is known about the immune responses of the much larger population of children with asymptomatic or mild symptomatic SARS-CoV-2 infection.

In this study, we evaluated the temporal evolution of SARS-CoV-2–specific humoral immune responses in a cohort of children and adolescents with asymptomatic or mildly symptomatic SARS-CoV-2 infection. We report clinical characteristics, antibody binding magnitude to a panel of SARS-CoV-2 antigens, and virus-neutralizing antibody responses over a period of up to 4 months after acute infection. Further, we compare the antibody responses of these children and adolescents to those of a group of adults with mild symptomatic SARS-CoV-2 infection. Knowledge of the effectiveness and durability of SARS-CoV-2–specific immune responses in children is critical to the development of pediatric vaccination strategies and approaches to mitigate SARS-CoV-2 transmission in schools and other congregate childcare settings.

## Results

### Patient characteristics.

We evaluated SARS-CoV-2–specific humoral immune responses in 69 children and adolescents (<21 years of age) who had participated in a study of acute SARS-CoV-2 infection (6). Clinical data and sera were collected from these participants during the acute phase of infection and approximately 2 and 4 months after acute infection. Median age was 11.5 years (IQR, 5.2–16.5 years), and 51% of participants were female (Table 1). The most common chronic medical conditions were obesity (29%) and asthma (6%). Fifty-five (80%) participants reported 1 or more symptoms associated with acute SARS-CoV-2 infection with a median symptom duration of 3 days (IQR, 2–7 days). The most common symptoms reported were fever (49%), cough (36%), and headache (29%). The remaining 14 (20%) participants reported no symptoms associated with acute infection (2 weeks before SARS-CoV-2 diagnosis and up to 28 days after study enrollment) and were classified as asymptomatic. Sera from acute infection were collected at a median of 9 days (IQR, 6–12 days) from symptom onset or SARS-CoV-2 diagnosis in asymptomatic participants. Sera from 2 months and 4 months after acute infection were collected at a median of 57 days (IQR, 50–71 days) and 109 days (IQR, 101–120 days) from symptom onset or SARS-CoV-2 diagnosis, respectively. To compare humoral immune responses across the full spectrum of age, we similarly evaluated humoral immune responses to SARS-CoV-2 in 24 adults (21 years of age or older). Median age was 43 years (IQR, 31–57 years), and 58% of participants were female (Table 1). All adult participants reported 1 or more symptoms associated with acute SARS-CoV-2 infection. No pediatric or adult individuals required hospitalization for SARS-CoV-2 infection.

### SARS-CoV-2–specific antibodies in sera from children and adolescents.

**Figure 1:**
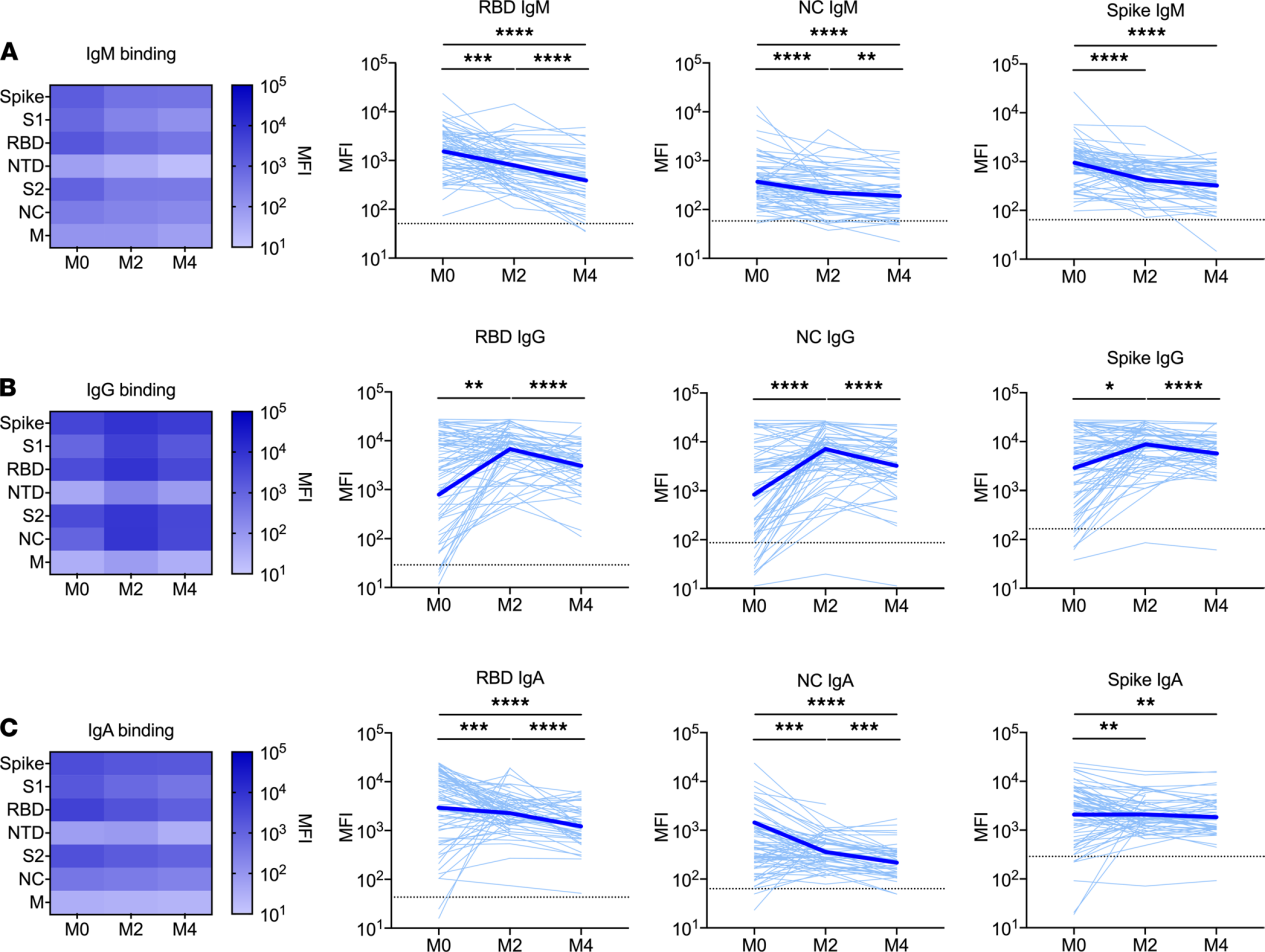
SARS-CoV-2–specific binding antibodies in children and adolescents after acute infection. Specific binding to SARS-CoV-2 antigens was measured by Luminex-based multiplex assays for IgM (**A**), IgG (**B**), and IgA (**C**) antibodies against the whole spike, subunit 1 (S1), receptor-binding domain (RBD), N-terminal domain (NTD), subunit 2 (S2), nucleocapsid (NC), and membrane (M) proteins. Binding is expressed as mean fluorescence intensity (MFI) of sera at the time of acute infection (M0) and 2 months (M2) and 4 months (M4) after acute infection. Heatmaps show binding to all analyzed SARS-CoV-2 antigens, with darker colors corresponding to higher binding. Line graphs depict RBD-, NC-, and whole spike–specific binding of sera from individual participants (light blue lines) and the geometric mean of all individuals (thick blue lines). Dotted lines indicate assay positivity thresholds and were calculated as the mean MFI plus 3 standard deviations using sera from 10 SARS-CoV-2–uninfected individuals. Comparisons of samples from individuals across time points were made using Wilcoxon signed-rank tests. *P < 0.05; **P < 0.01; ***P < 0.005; ****P < 0.0001.

We used Luminex-based binding antibody multiplex assays to measure SARS CoV-2–specific antibodies targeting whole spike, subunit 1 (S1), receptor-binding domain (RBD), N-terminal domain (NTD), subunit 2 (S2), nucleocapsid (NC), and membrane (M) proteins in sera from children and adolescents during acute infection (*n* = 69), 2 months after acute infection (*n* = 56), and 4 months after acute infection (*n* = 50). At the time of acute infection, all children and adolescents had detectable levels of IgM and IgG antibodies against 1 or more SARS-CoV-2 antigens, and 68 (99%) had a detectable IgA response to 1 or more SARS-CoV-2 antigens. Levels of SARS-CoV-2–specific IgM and IgA antibodies were highest during acute infection and declined at 2 and 4 months after acute infection (Figure 1, Supplemental Figure 1, and Supplemental Table 1; supplemental material available online with this article; https://doi.org/10.1172/jci.insight.150909DS1). Notably, NC-specific IgA antibodies appeared to decline somewhat more rapidly than IgA antibodies targeting other antigens, including portions of the spike protein (Figure 1, A–C and Supplemental Figure 1C); however, IgA and IgM antibodies against most antigens were still detected in the majority of children 4 months after acute infection. In contrast, levels of IgG antibodies generally increased from acute infection to 2 months after infection before decreasing between 2 and 4 months after acute infection. We also measured levels of IgG subclasses against SARS-CoV-2 antigens at 0, 2 and 4 months after acute infection (Supplemental Figure 2A). Between acute infection and 2 months after acute infection, we observed significant increases in the ratios of IgG1/IgG3 antibodies against spike (*P* = 0.04), S1 (*P* < 0.0001), RBD (*P* < 0.0001), S2 (*P* = 0.03), and NC (*P* < 0.0001); these differences persisted when antibody levels against the same antigens were compared between acute infection and 4 months after acute infection (spike, *P* < 0.0001; S1, *P* < 0.0001; RBD, *P* < 0.0001; S2, *P* < 0.0001; NC, *P* < 0.0001). No differences in the ratios of IgG1/IgG3 antibodies against NTD or M were observed over time (Supplemental Figure 2B).

### SARS-CoV-2–infected children and adolescents generate durable neutralizing antibody responses.

**Figure 2:**
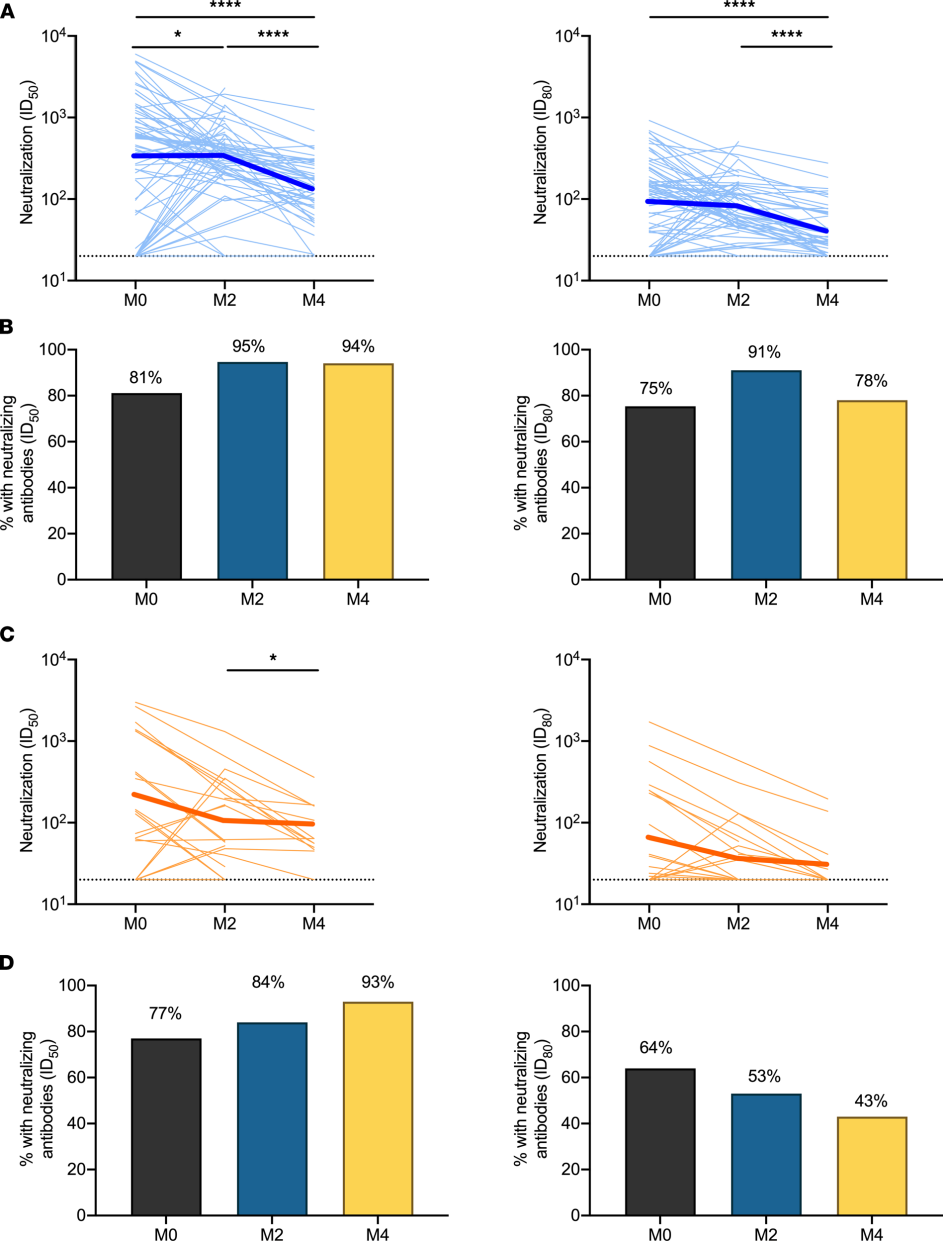
SARS-CoV-2–neutralizing antibodies in sera from children, adolescents, and adults. Antibody-mediated neutralization activity was measured using a pseudovirus (614G) assay and is presented as 50% inhibitory dilution (ID_50_) or 80% inhibitory dilution (ID_80_). Neutralization activity in sera from specific individuals (light blue and light orange lines) and the geometric mean of all individuals (thick blue and thick orange lines) is shown at the time of acute infection (M0) and 2 months (M2) and 4 months (M4) after acute infection in children and adolescents (**A**) and adults (**C**). Dotted lines indicate assay positivity thresholds. Comparisons of samples from individuals across time points were made using Wilcoxon signed-rank tests. Proportion of children and adolescents (**B**) and adults (**D**) with detectable neutralizing antibodies at ID_50_ (left) and ID_80_ (right) at specific time points after acute infection. *P < 0.05; ****P < 0.0001.

We next measured neutralizing activity of sera from children, adolescents, and adults using a luciferase-based (Luc-based) SARS-CoV-2 pseudovirus (614G) assay, as previously described (23). Among children and adolescents, neutralizing antibodies at a 50% inhibitory dilution (ID_50_) were detected in 56 of 69 (81%) serum samples collected during acute infection, 53 of 56 (95%) samples collected 2 months after acute infection, and 47 of 50 (94%) of samples collected 4 months after acute infection (Figure 2, A and B). Acute infection samples for which there was no appreciable neutralizing activity were generally collected earlier after symptom onset or SARS-CoV-2 diagnosis than acute infection samples with detectable neutralizing antibodies (median, 7 days [IQR, 5–8 days] vs. 9 days [IQR, 7–13 days]; *P* = 0.01]. Between acute infection and 2 months after acute infection, levels of neutralizing antibodies declined in 31 (55%) children, increased in 24 (43%) children, and were stable in 1 (2%) child (median ID_50_, 577 vs. 379; *P* = 0.04). Neutralizing antibodies declined in 31 of 37 (84%) children with paired samples at 2 and 4 months after acute infection (median ID_50_, 369 vs. 174; *P* < 0.0001). Among adults, virus-neutralizing activity at ID_50_ was detected in 17 of 22 (77%) serum samples collected during acute infection, 16 of 19 (84%) samples collected at 2 months after acute infection, and 13 of 14 (93%) samples collected at 4 months after acute infection (Figure 2, C and D). As observed among children and adolescents, acute infection samples for which there was no appreciable neutralizing activity were collected earlier after symptom onset than acute infection samples with detectable neutralizing antibodies (median, 4 days [IQR, 3–8 days] vs. 16 days [IQR, 12–20 days]; *P* = 0.004]. As anticipated, neutralizing activity measured in the pseudovirus assay was most strongly correlated with levels of antibodies against spike, S1, and RBD (Supplemental Figure 3). Further, during the acute phase of infection, we noted a robust correlation between neutralization activity and levels of IgM and IgA antibodies targeting spike antigens, though IgG levels correlated more closely with neutralization than IgM or IgA levels at later time points (Supplemental Figure 3), suggesting that the contribution of specific antibody isotypes to neutralization activity may vary at different times points after infection.

### SARS-CoV-2–specific antibody responses are similar in asymptomatic and mildly symptomatic children.

**Figure 3:**
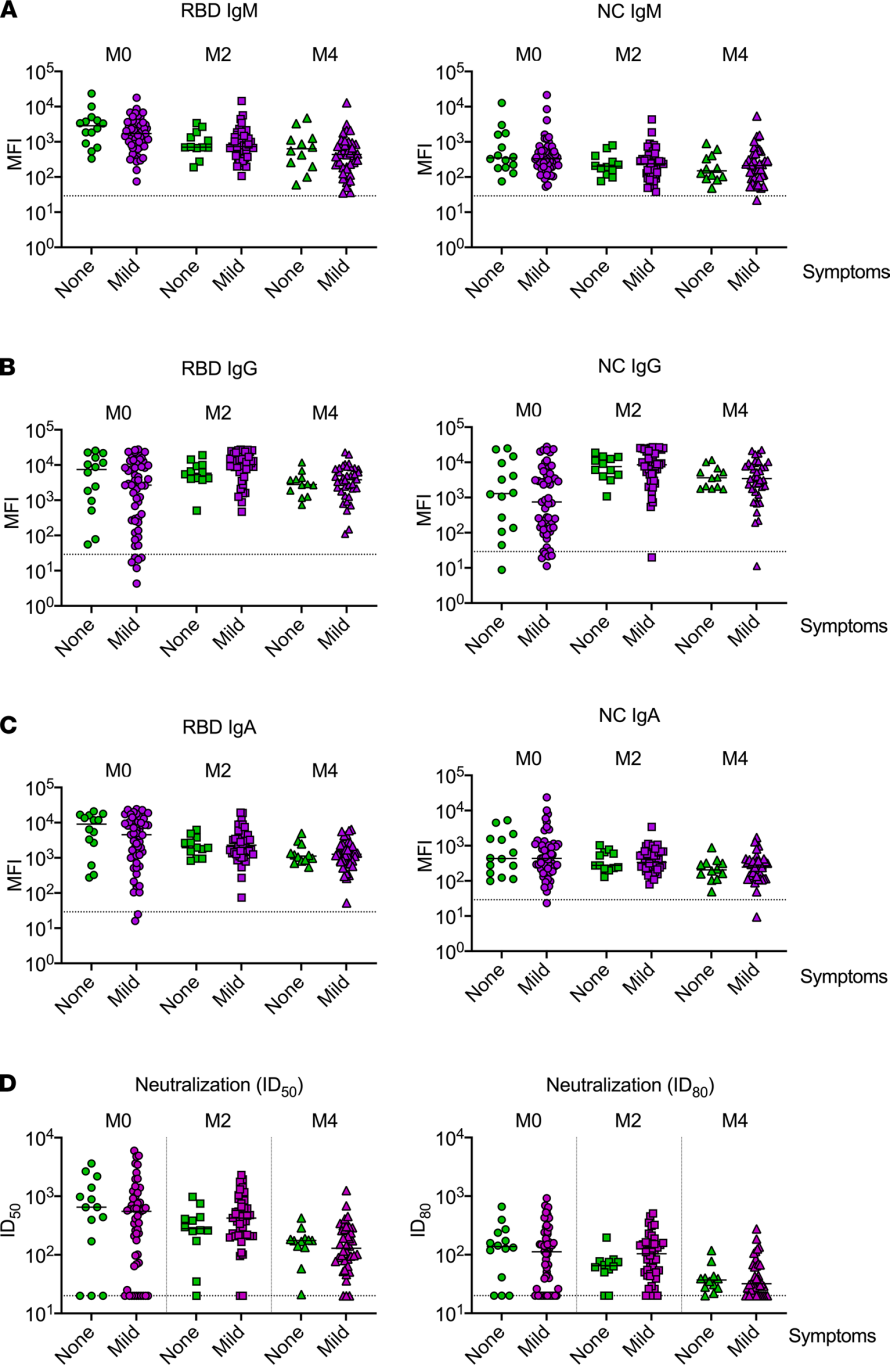
SARS-CoV-2–specific antibodies and neutralizing activity in sera from children and adolescents with asymptomatic compared with mild symptomatic infection. Levels of IgM (**A**), IgG (**B**), and IgA (**C**) antibodies against SARS-CoV-2 receptor-binding domain (RBD) and nucleocapsid (NC) proteins were measured by Luminex-based multiplex assays and are expressed as mean fluorescence intensity (MFI). Binding is shown at the time of acute infection (M0) and 2 months (M2) and 4 months (M4) after acute infection. Dotted lines for binding assays correspond to the mean MFI plus 3 standard deviations in sera from 10 SARS-CoV-2–uninfected individuals. (**D**) Antibody-mediated neutralization activity was measured using a pseudovirus (614G) assay and is presented as 50% inhibitory dilution (ID_50_) or 80% inhibitory dilution (ID_80_). No significant differences in levels of antibodies against specific SARS-CoV-2 antigens or neutralizing activity were seen at any time point among children with asymptomatic versus mild symptomatic infection (Wilcoxon rank-sum tests).

Given that prior studies in adults suggested that humoral immune responses to SARS-CoV-2 infection may differ based on symptom severity (5, 24–29), we compared antibody responses among children and adolescents with asymptomatic and mild symptomatic SARS-CoV-2 infection. We observed no significant differences in the levels of IgM, IgG, or IgA antibodies specific to any SARS-CoV-2 antigen during acute infection or at 2 or 4 months after acute infection in asymptomatic and mildly symptomatic children (Figure 3, A–C). Similarly, the degree of pseudovirus neutralization activity in sera did not differ based on the presence of symptoms at any of these time points (Figure 3D).

### Comparisons of SARS-CoV-2 antibody responses in children, adolescents, and adults.

**Figure 4:**
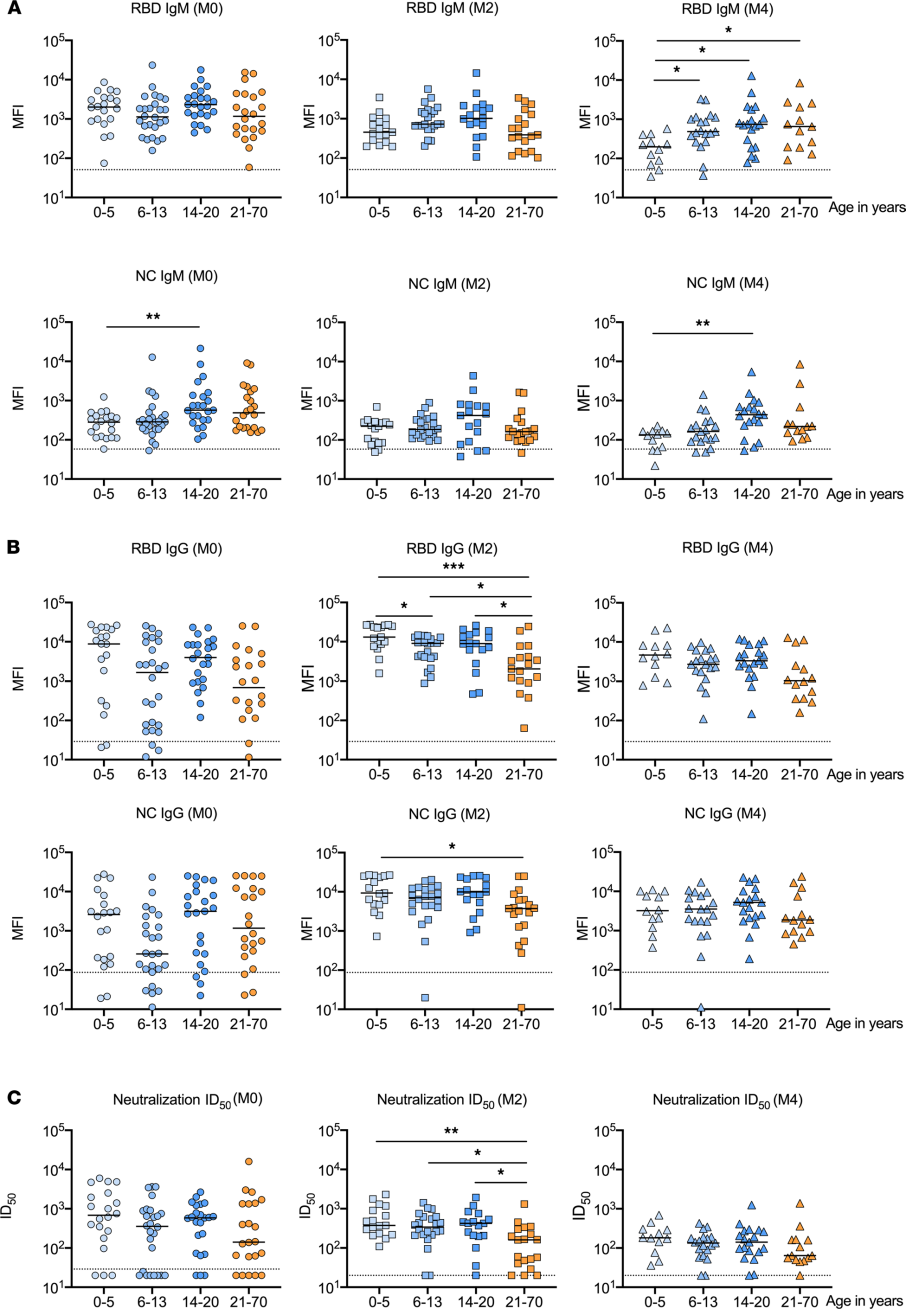
SARS-CoV-2–specific antibodies and neutralizing activity in sera from children and adults by age. Levels of IgM (**A**) and IgG (**B**) antibodies against SARS-CoV-2 receptor-binding domain (RBD) and nucleocapsid (NC) proteins were measured by Luminex-based multiplex assays and are expressed as mean fluorescence intensity (MFI). Dotted lines for binding assays correspond to the mean MFI plus 3 standard deviations in sera from 10 SARS-CoV-2–uninfected individuals. (**C**) Antibody-mediated neutralization activity was measured in a pseudovirus (614G) assay and is presented as 50% inhibitory dilution (ID_50_). Dotted lines for neutralization assays correspond to the assay threshold. Comparisons of antibody measurements by age category were performed at acute infection (M0) and 2 months (M2) and 4 months (M4) after acute infection using Wilcoxon rank-sum tests and adjusted for multiple comparisons using the Benjamini-Hochberg procedure. *P < 0.05; **P < 0.01; ***P < 0.005.

We next compared the SARS-CoV-2 humoral immune responses of children and adolescents to those of adults. IgM and IgG antibodies against at least 1 SARS-CoV-2 antigen were detected in all adults at each time point; IgA antibodies were found in 22 of 22 (100%) samples collected during acute infection, 18 of 19 (95%) samples collected at 2 months after acute infection, and 11 of 14 (79%) samples collected at 4 months after acute infection (Supplemental Figure 4 and Supplemental Table 2). In order to evaluate for age-related differences in humoral immune responses to SARS-CoV-2, we compared levels of SARS-CoV-2–specific antibodies in children, adolescents, and adults at each time point. RBD-specific IgM titers were largely similar across age groups during acute infection and 2 months after acute infection (Figure 4A); however, levels of RBD IgM antibodies declined more quickly in children 0–5 years of age and differed from all other age groups at 4 months after acute infection. NC-specific IgM titers tended to be lower in children 0–5 years of age or 6–13 years of age at all time points. Similar trends were observed by age for IgM antibodies against other SARS-CoV-2 antigens with the exception of M protein, as adults had significantly lower levels of M-specific IgM antibodies than children or adolescents at all time points (Supplemental Figure 5A). Levels of RBD-specific IgG were generally similar across age groups during acute infection (Figure 4B), but all children, regardless of age group, had higher levels of RBD IgG than adults at 2 months (median mean fluorescence intensity [MFI], 9360 [IQR, 4520–14451] vs. 2028 [IQR, 965–4022]; *P* = 0.0001) and 4 months after acute infection (median MFI, 3494 [IQR, 1941–6721] vs. 1043 [IQR, 414–2379]; *P* = 0.02). Similar differences by age across these time points were observed for IgG antibodies specific to NC (Figure 4B) and other SARS-CoV-2 antigens (Supplemental Figure 5B). Levels of IgA antibodies against spike, S2, and NC were generally lower among children 0–5 years or 6–13 years of age than among older age groups at all time points (Supplemental Figure 5C). Neutralizing activity in sera was similar across age groups at the time of acute infection. However, paralleling the trends observed for SARS-CoV-2–specific IgG antibodies, children and adolescents tended to have higher neutralizing activity than adults at 2 months (median ID_50_, 379 [IQR, 214–634] vs. 161 [IQR, 44–262]; *P* = 0.0003) and 4 months after acute infection (median ID_50_, 148 [IQR, 81–254] vs. 64 [IQR, 52–162]; *P* = 0.10), although these differences were not statistically significant at 4 months (Figure 4C).

## Discussion

In this study, we describe the durability and functionality of the humoral immune responses of children and adolescents with asymptomatic or mild symptomatic SARS-CoV-2 infection. SARS-CoV-2 infection elicited robust neutralizing antibody responses during acute infection and up to 4 months after acute infection in children and adolescents. Moreover, these antibody responses did not differ among children with mild symptomatic infection compared with those with asymptomatic infection, suggesting that effective humoral responses are elicited regardless of the severity of the acute infection. Notably, we found that the SARS-CoV-2–specific antibody responses of children and adolescents were generally more robust and durable than those of adults with mild symptomatic infection.

Several prior studies evaluated the prevalence and durability of SARS-CoV-2 infection–induced humoral immune responses in adult populations. Studies conducted early in the COVID-19 pandemic reported that seroconversion occurs in approximately 90%–95% of SARS-CoV-2–infected adults, with SARS-CoV-2–specific IgM, IgG, and IgA antibodies detectable in most individuals within 2 weeks of symptom onset (2, 4, 30–34). Similarly, we found that 100% of children in our study exhibited IgM and IgG responses and 99% exhibited IgA responses within this time period. Multiple reports have demonstrated that SARS-CoV-2–specific antibody responses among adults are durable, with a recent study reporting that IgG to the SARS-CoV-2 spike protein is detectable at least 6 months after infection in more than 90% of adults (3). Neutralizing antibody responses, which are believed to correlate most closely with protection from reinfection (35, 36), are reportedly stable for at least 3 months after infection in adults (37, 38). Similarly, seroconversion occurred in all children and adolescents evaluated in our study. Neutralizing activity was more likely to be detectable in sera collected from both child and adult participants who were slightly further into the course of their acute infection, indicating a slight lag in the development of an effective neutralizing antibody response. Importantly, SARS-CoV-2–specific humoral immune and virus-neutralizing responses were detectable in the vast majority of individuals 4 months after acute infection, demonstrating that neutralizing antibody responses in children are similar in duration to those in adults.

Recent studies in adults have described differences in the durability of IgA, IgM, and IgG antibody responses to SARS-CoV-2. Glück and colleagues conducted serial serological assays in sera from 123 SARS-CoV-2–infected adults over a 30-week period (39). They found that IgM and IgA responses waned more rapidly than IgG, with 90% of participants having detectable levels of IgG but only 25% of participants having detectable levels of IgA and IgM at the end of the study. Sterlin and colleagues reported a similar trend in 159 adults hospitalized for COVID-19, with IgA antibodies dominating early responses to the virus (~3 weeks after symptom onset), followed by rapid waning of IgA antibody levels and a marked increase in IgG levels (40). We observed higher IgG levels compared with IgA or IgM levels at 4 months after acute infection; however, IgA, IgM, and IgG responses were detected in the majority of participants from all age categories at this time point, with few significant differences observed across age groups. In addition to differential waning of IgM, IgG, and IgA responses to SARS-CoV-2, several groups have reported associations between antibody type and neutralization response. In the aforementioned study by Sterlin and colleagues, serum IgA contributed to virus-neutralizing activity to a greater extent than serum IgG during the first month after symptom onset (40). Moreover, Harrington and colleagues observed an association between anti–S trimer IgM and neutralization activity in 34 adults with mild COVID-19 (41). We observed a slightly stronger correlation between IgM antibody levels and neutralization activity compared with the correlation between IgG and IgA antibody levels and neutralization activity during the acute phase of infection, similarly suggesting that IgM could contribute to viral neutralization early after infection in children. However, in order to definitively delineate the contribution of different immunoglobulin isotypes to SARS-CoV-2 neutralization, it will be necessary to directly compare the neutralization activity of purified isotype-specific antibodies.

To date, most studies of the immune responses of children to SARS-CoV-2 have been cross-sectional, have assessed immunity only during acute infection, or have focused on patients hospitalized for severe COVID-19 or MIS-C (22). Weisberg and colleagues evaluated SARS-CoV-2–specific antibody levels and neutralizing activity in nonhospitalized adults with COVID-19, hospitalized adults with COVID-19 acute respiratory distress syndrome, children with MIS-C, and SARS-CoV-2–infected children, half of whom were asymptomatic (18). They found no significant differences in antibody levels or neutralizing activity between the two groups of children; however, both groups of adults exhibited higher SARS-CoV-2–specific antibody levels and neutralizing activity than either group of children (18). Similarly, Pierce and colleagues found that children who were hospitalized with COVID-19 or MIS-C had lower levels of neutralizing activity than hospitalized adults (21). In our study, we found that SARS-CoV-2–specific antibody levels and virus-neutralizing activity among children and adolescents with asymptomatic or mild symptomatic SARS-CoV-2 infection were generally similar to those of adults with mild symptomatic infection. Importantly, levels of SARS-CoV-2–specific IgG and serum neutralizing activity were similar at the time of acute infection but generally higher in children and adolescents than adults at 2 and 4 months after acute infection, suggesting that SARS-CoV-2–specific IgG responses may decline more slowly in children and adolescents. In contrast, we observed higher levels of SARS-CoV-2–specific IgM and IgA antibodies with increasing age, particularly 4 months after acute infection, suggesting that young children generate less robust and durable responses for these SARS-CoV-2–specific antibody isotypes. Overall, our findings indicate that children and adolescents have a similar degree of protective immunity as adults after mild or asymptomatic SARS-CoV-2 infection. Given similarities in the response to natural infection in children and adults, it is likely that vaccination against SARS-CoV-2 will also elicit a similar degree of protection across the full spectrum of age, as has recently been reported for the Pfizer-BioNTech vaccine in children 12–15 years of age (42). Though we cannot directly compare our results to the neutralizing antibody titers reported in vaccine trial studies, both the vaccine trial data and our results suggest that younger age may be associated with greater neutralizing antibody responses. Importantly, both the vaccine trial results and our data demonstrated the ability of children to elicit robust SARS CoV-2–specific antibody responses. Future studies will need to directly evaluate associations between age and SARS-CoV-2 vaccine responses.

There are conflicting data regarding the impact of illness severity on immune responses to SARS-CoV-2 infection. Lau and colleagues found that the neutralizing activity of sera from 195 adults and children with prior SARS-CoV-2 infection was correlated with disease severity, as classified by presence of symptoms and need for supplemental oxygen (24). Similarly, Dogan and colleagues reported a positive correlation between level of care required and both elevated SARS-CoV-2–specific antibody levels and neutralization activity in a study of 115 adult participants (25). Supporting this finding, several other groups reported lower humoral immune responses in patients with mild or asymptomatic disease, including a complete lack of detectable levels of circulating SARS-CoV-2–specific immunoglobulins in some cases (26–29). In contrast, we observed similar levels of SARS-CoV-2–specific IgM, IgG, and IgA antibodies and neutralizing activity up to 4 months after acute infection in asymptomatic and mildly symptomatic children. Given that severe illness is uncommon in SARS-CoV-2–infected children and adolescents, our findings indicate that the vast majority of children and adolescents with SARS-CoV-2 infection can be anticipated to generate robust and durable immune responses to the virus.

While our study provides potentially novel insights into antibody responses to SARS CoV-2 in children, we acknowledge that this study has some limitations. First, we focused on pediatric and adult populations with asymptomatic and mild infections and, specifically, did not include participants with severe COVID-19 or children with MIS-C; thus, our findings are generalizable only to individuals with asymptomatic or mild symptomatic SARS-CoV-2 infection. Second, we did not evaluate cellular immunity, which is likely required for long-term immunological memory, or systemic inflammatory responses, which may contribute to the establishment of immune memory (3). Third, while several studies in adults have measured antibody responses out to 8 months after acute infection, we currently only have data for children and adolescents up to 4 months after infection. Finally, additional studies are needed to evaluate the impact of emerging SARS-CoV-2 variants on viral-specific immune responses in children and adolescents and to evaluate if immune responses induced by prior infection provide broad protection from infection by these variants.

In conclusion, we found that children and adolescents with asymptomatic or mild symptomatic SARS-CoV-2 infection mount broad, effective, and durable antibody responses that exhibit robust viral neutralizing activity at least 4 months after acute infection. Notably, these responses were largely similar or superior to those observed in adults with symptomatic SARS-CoV-2 infection who did not require hospitalization. Our findings suggest that children and adolescents develop effective humoral immune responses irrespective of illness severity that are likely to contribute to protection against reinfection, thereby contributing to the establishment of herd immunity.

## Methods

### Study design.

The Duke Biospecimens from RespirAtory Virus-Exposed Kids (BRAVE Kids) study is a prospective cohort study of children and adolescents (<21 years of age) with confirmed SARS-CoV-2 infection or close contact with an individual with confirmed SARS-CoV-2 infection (6).

### Study procedures.

The analyses presented herein were limited to participants with SARS-CoV-2 infection diagnosed by PCR between May 1, 2020, and July 31, 2020. SARS-CoV-2 was detected from nasopharyngeal or nasal swabs through PCR testing performed for clinical care or through a quantitative real-time PCR assay, as previously described (6). We collected exposure, sociodemographic, and clinical data at the time of enrollment through review of electronic medical records and a directed caregiver questionnaire conducted by telephone. In addition, we conducted a home visit to collect whole blood from participants via venipuncture and to obtain other biospecimens. Serum was isolated from whole blood via centrifugation and frozen to –80°C before analysis. We conducted follow-up study visits at home or at a research clinic site approximately 2 and 4 months after acute infection.

### Adult study participants.

We obtained previously collected sera from participants enrolled in the Duke University Molecular and Epidemiological Study of Suspected Infection (MESSI) (43). Participants in this prospective cohort are identified via community-enrollment, the Duke University Health System (DUHS), or the Durham Veterans Affairs Health System (DVAHS) as having SARS-CoV-2 infection by PCR testing performed either at the North Carolina State Laboratory of Public Health or through the clinical laboratories at either DUHS or DVAHS. Participants were sampled between March and December 2020. Mild disease was defined as any PCR-confirmed infection that did not require hospitalization.

### Measurement of serum SARS-CoV-2–specific antibodies.

We measured serum antibodies using a customized binding antibody multiplex assay against the following SARS-CoV-2 antigens: whole spike (SinoBiological, 40589-V08B1), S1 (SinoBiological, 40591-V08H), S2 (SinoBiological, 40590-V08B), RBD (SinoBiological, 40592-V08H), NTD (SinoBiological, 40591-V49H), NC (SinoBiological, 40588-V08B), and M (MyBiosource, MBS8574735) proteins. SARS-CoV-2 antigens were covalently coupled to magnetic fluorescent beads (MagPlex biospheres, Luminex). Unconjugated (blank) beads were included to monitor nonspecific binding. After a pilot assay to identify the optimal serum dilution, antigen-coupled beads were incubated with a 1:400 serum dilution for measurement of IgG and IgG subclasses and 1:100 serum dilution for measurement of IgA and IgM. Antibody binding to the bead-coupled antigens was then detected with PE-conjugated mouse anti-human IgM (Southern Biotech, 9020-09) and IgG (Southern Biotech, 9040-09) or PE-conjugated goat anti-human IgA (Southern Biotech, 2050-09) at 2 μg/ml, using a Bio-Plex 200 instrument (Bio-Rad Laboratories), which rendered a MFI for each sample. For measurement of SARS CoV-2–specific IgG1 and IgG3, a biotinylated mouse anti-human IgG-1 or IgG-3 followed by PE-conjugated streptavidin was used for detection. Sera from 10 individuals collected before the COVID-19 pandemic (2013–2014) was used to define the assay positivity threshold for each antigen (mean MFI plus 3 standard deviations). A prescreened pooled serum sample of 2 unrelated SARS-CoV-2–infected donors was used as positive control in all assays to ensure reproducibility between assays and to ensure detection of antibodies against all antigens tested. Criteria for accepting results included equal to or less than 20% coefficient of variation of the 2 duplicates with a bead count of equal to or more than 100 for each sample.

### SARS-CoV-2 pseudovirus neutralization assays.

SARS-CoV-2 neutralization was measured with spike-pseudotyped viruses in HEK-293T-hACE2 cells as a function of reduction in Luc reporter activity. We used an *env*-deficient HIV-based lentiviral system to produce viral particles pseudotyped with SARS-CoV-2 spike. An expression plasmid encoding the codon-optimized full-length spike protein of the Wuhan-1 strain (VRC7480) was provided by the Vaccine Research Center at the NIH. The D614G amino acid change was introduced into VRC7480 by site-directed mutagenesis using the QuikChange Lightning Site-Directed Mutagenesis Kit (Agilent Technologies) and confirmed by full-length spike gene sequencing. Pseudovirions were produced in HEK 293T/17 cells (ATCC) by using FuGene 6 (Promega) and cotransfecting spike plasmid, lentiviral backbone plasmid (pCMV ΔR8.2), a firefly Luc reporter gene plasmid (pHR’ CMV Luc) (44), and a plasmid containing TMPRSS2 (required for cell entry) (plasmids provided by David Montefiori, Duke University School of Medicine). TCID_50_ assays were performed on thawed pseudovirus aliquots using HEK-293T-hACE2 cells (BEI Resources) to determine the infectious dose for neutralization assays.

For neutralization, serum samples were heat inactivated for 30 minutes at 56°C before testing. Samples were serially diluted 5-fold to 8 points in duplicate and incubated with a dose of pretitrated pseudovirus for 1–1.5 hours at 37°C in 96-well flat-bottom poly-L-lysine–coated culture plates (Corning Biocoat). HEK-293T-hACE2 cells were suspended using TrypLE Select Enzyme solution (Thermo Fisher Scientific) and immediately added to all wells (10,000 cells in 100 μL of growth medium per well). One set of 8 control wells received cells plus virus (virus control) and another set of 8 wells received cells only (background control). After 72 hours of incubation, medium was removed and 30 μL Promega 1X lysis buffer was added. After a 10-minute incubation at room temperature, 100 μl Bright-Glo Luc reagent (Promega) was added to all wells. After 2 minutes, 110 μl of the cell lysate was transferred to a black/white plate. Luminescence was measured using a PerkinElmer Life Sciences model Victor3 luminometer. Neutralization titers represent the serum dilution at which relative luminescence units (RLUs) were reduced by either 50% (ID_50_) or 80% (ID_80_) compared with virus control wells after subtraction of background RLUs.

### Statistics.

We described characteristics of the study population using frequencies and percentages for categorical variables and medians and IQRs for continuous variables. We used χ^2^ or Fisher’s exact tests for comparisons of categorical variables. To compare humoral immune responses at specific time points based on the presence of symptoms and across age categories, we used Wilcoxon rank-sum tests or 2-way ANOVA based on Gaussian distribution of a variable. To account for repeated measurements from individuals, we used Wilcoxon signed-rank tests to compare humoral immune responses in paired serum samples collected from individuals across specific time points. We adjusted for multiple comparisons using the Benjamini-Hochberg procedure (45). Study data were managed using REDCap electronic data capture tools hosted at Duke University (46). Analyses were performed using GraphPad Prism and R version 4.0.3 (47).

### Study approval.

The BRAVE Kids study is being conducted within the DUHS in Raleigh-Durham, North Carolina, USA. DUHS is a large, integrated health system consisting of 3 hospitals and over 100 outpatient clinics. This study was approved by the DUHS Institutional Review Board (Pro00106150). Informed consent was obtained from all study participants or their legal guardians, with written approval obtained using an electronic consent document. The MESSI study was approved by the DUHS Institutional Review Board (Pro00100241). Informed consent was obtained from all study participants, with written approval obtained using an electronic consent document.

## Author contributions

Conceptualization was done by CG, JHH, MSK, and GGF. Methodology was designed by CG, JHH, MSK, and GF. Investigation was performed by CG, JHH, CGL, JNA, JR, TSP, TS, ECS, DJL, ATR, NAT, TWB, MTM, EAP, SRP, MAM, CWW, MSK, and GGF. Writing of the original draft was performed by CG, JHH, MSK, and GGF. Review and editing of the draft were performed by CG, JHH, CGL, JNA, JR, TSP, TS, ECS, DJL, ATT, NAT, TWB, MTM, EAP, SRP, MAM, CWW, MSK, and GGF. Funding acquisition was done by JHH, SRP, CWW, MSK, and GGF. Resources were provided by SRP, MAM, CWW, MSK, and GGF. Project management was done by JHH. Supervision was provided by MSK and GGF.

## Supplementary Material

Supplemental data

## Figures and Tables

**Table 1:**
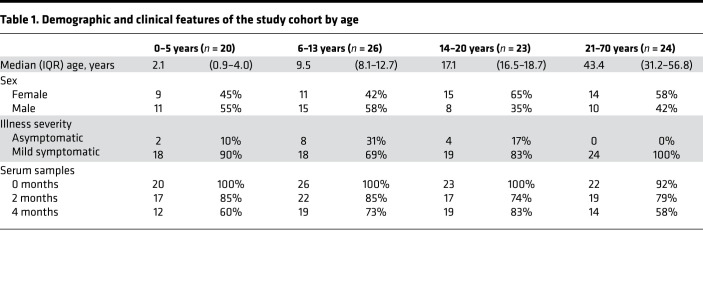
Demographic and clinical features of the study cohort by age

## Version 1 07/06/2021

In-Press Preview

## Version 2 09/08/2021

Electronic publication
