# Supplementary material for: Asymptomatic or mild symptomatic SARS-CoV-2 infection elicits durable neutralizing antibody responses in children and adolescents
Source: JCI Insight. 2021 Sep 8;6(17):e150909. doi: 10.1172/jci.insight.150909 (PMC8492306; doi:10.1172/jci.insight.150909)
Supplement: Supplemental data [file jciinsight-6-150909-s062.pdf]

Supplemental Figures

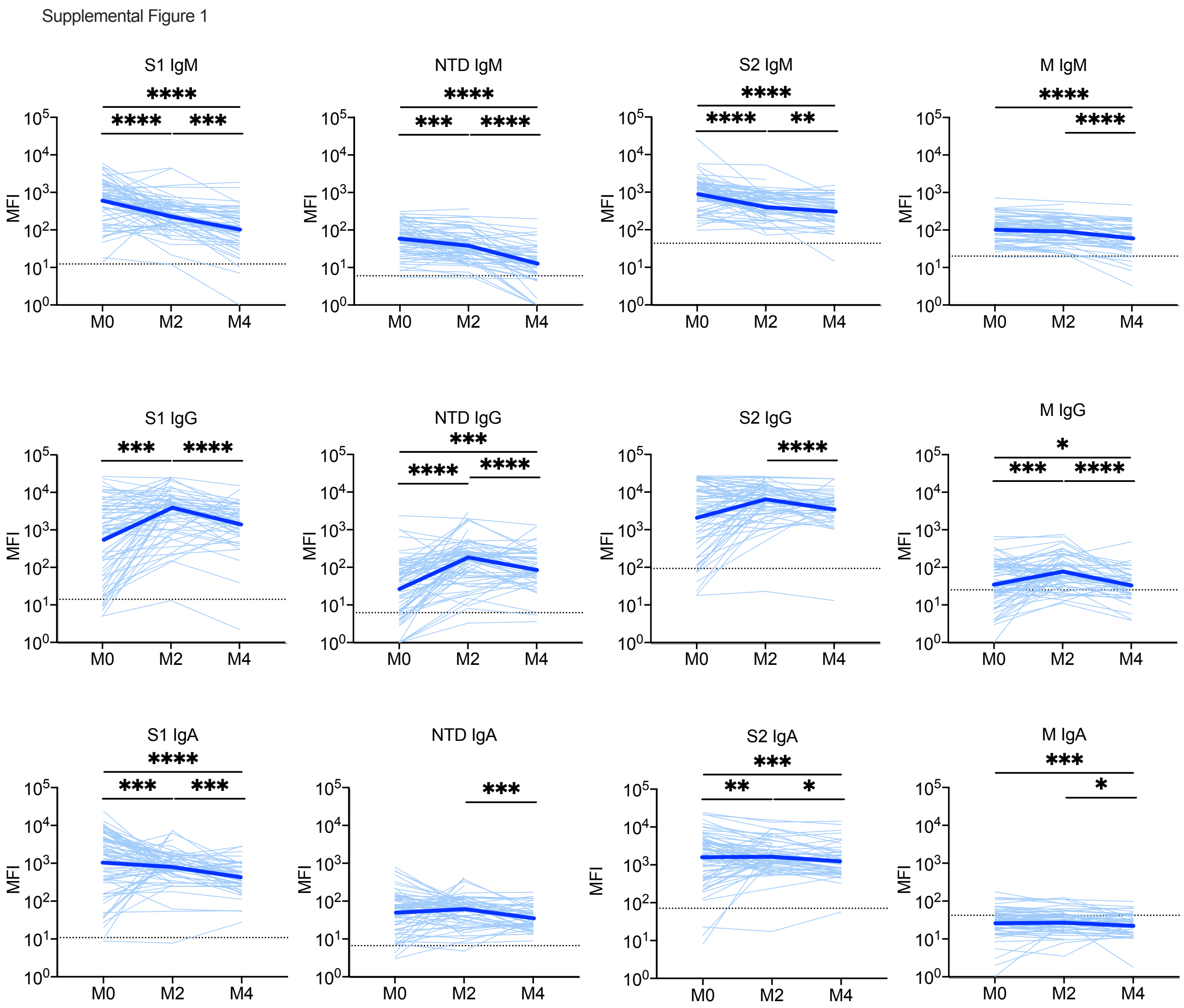

Supplemental Figure 1. SARS-CoV-2-specific binding antibodies in sera from children and adolescents. Binding to specific SARS-CoV-2 antigens including subunit 1 (S1), N-terminal domain (NTD), subunit 2 (S2), and membrane (M) proteins was measured by a Luminex-based multiplex assay for IgM (**A**), IgG (**B**), and IgA (**C**). Mean fluorescence intensity (MFI) of sera from specific individuals (light blue lines) and the geometric mean in all individuals (thick blue lines) are shown at the time of acute infection (M0) and 2 months (M2) and 4 months (M4) after acute infection. The dotted lines indicate assay thresholds corresponding to the mean MFI plus three standard deviations in sera from 10 SARS-CoV-2-

uninfected individuals. Levels of specific antibodies were compared across time points using Wilcoxon signed-rank tests. * $p<0.05$; ** $p<0.01$; *** $p<0.005$; **** $p<0.0001$

Supplemental Figure 2

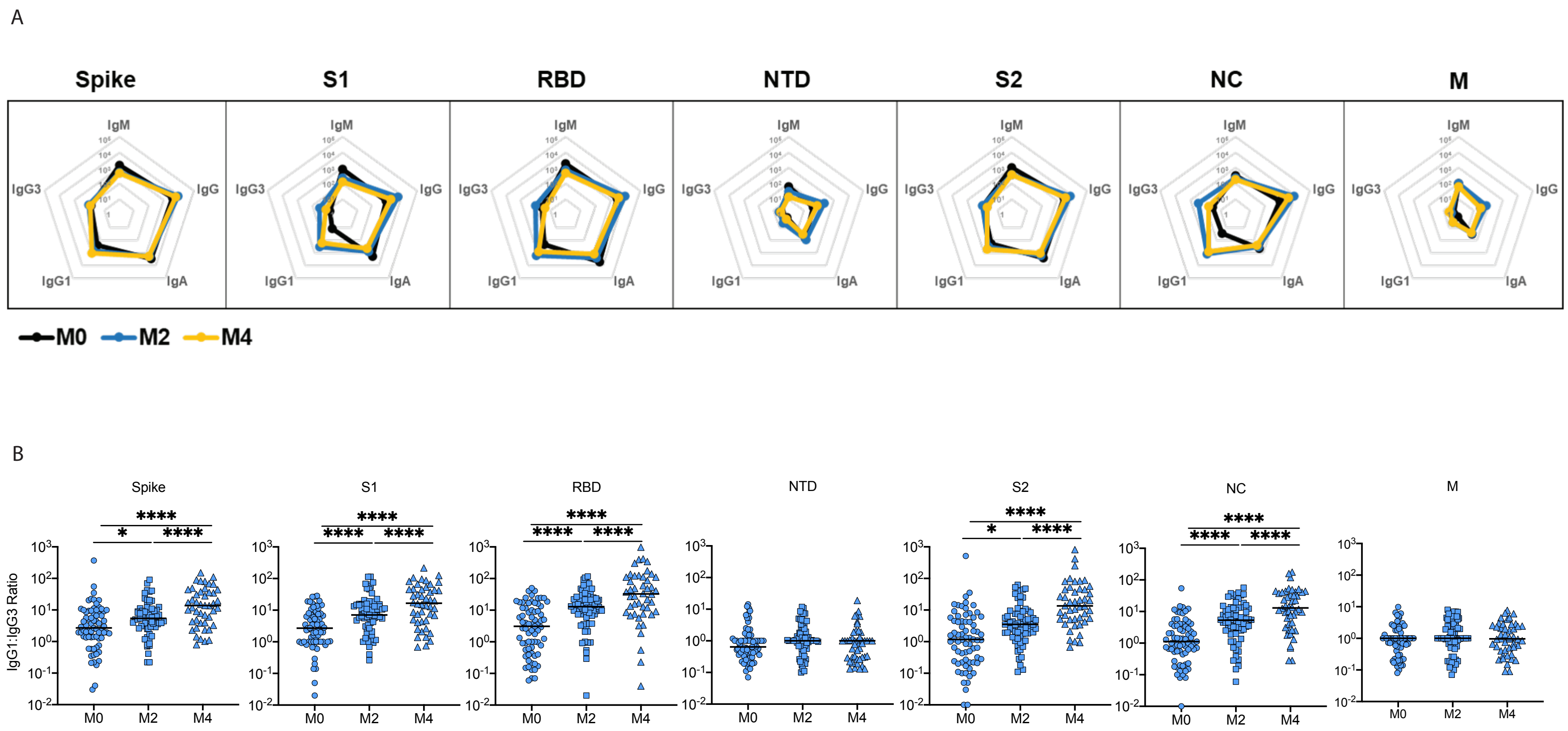

Supplemental Figure 2. SARS-CoV-2 antigen-specific antibody isotypes and IgG subclass binding. Antigen-specific binding to spike, subunit 1 (S1), receptor binding domain (RBD), N-terminal domain (NTD), subunit 2 (S2), nucleocapsid (NC), and membrane (M) proteins was measured using a Luminex-based multiplex assay for IgM, IgG, IgG1, IgG3, and IgA. The graphs depict (**a**) the distribution of each antibody isotype or subclass represented as mean fluorescence intensity (MFI) and (**b**) the IgG1:IG3 ratio for each of the antigens. Data are shown for

binding during the acute phase of infection (M0), 2 months after acute infection (M2), and 4 months after acute infection (M4). IgG1:IgG3 ratios were compared across time points using Wilcoxon signed-rank tests. * $p<0.05$; ** $p<0.01$; *** $p<0.005$; **** $p<0.0001$

Supplemental Figure 3

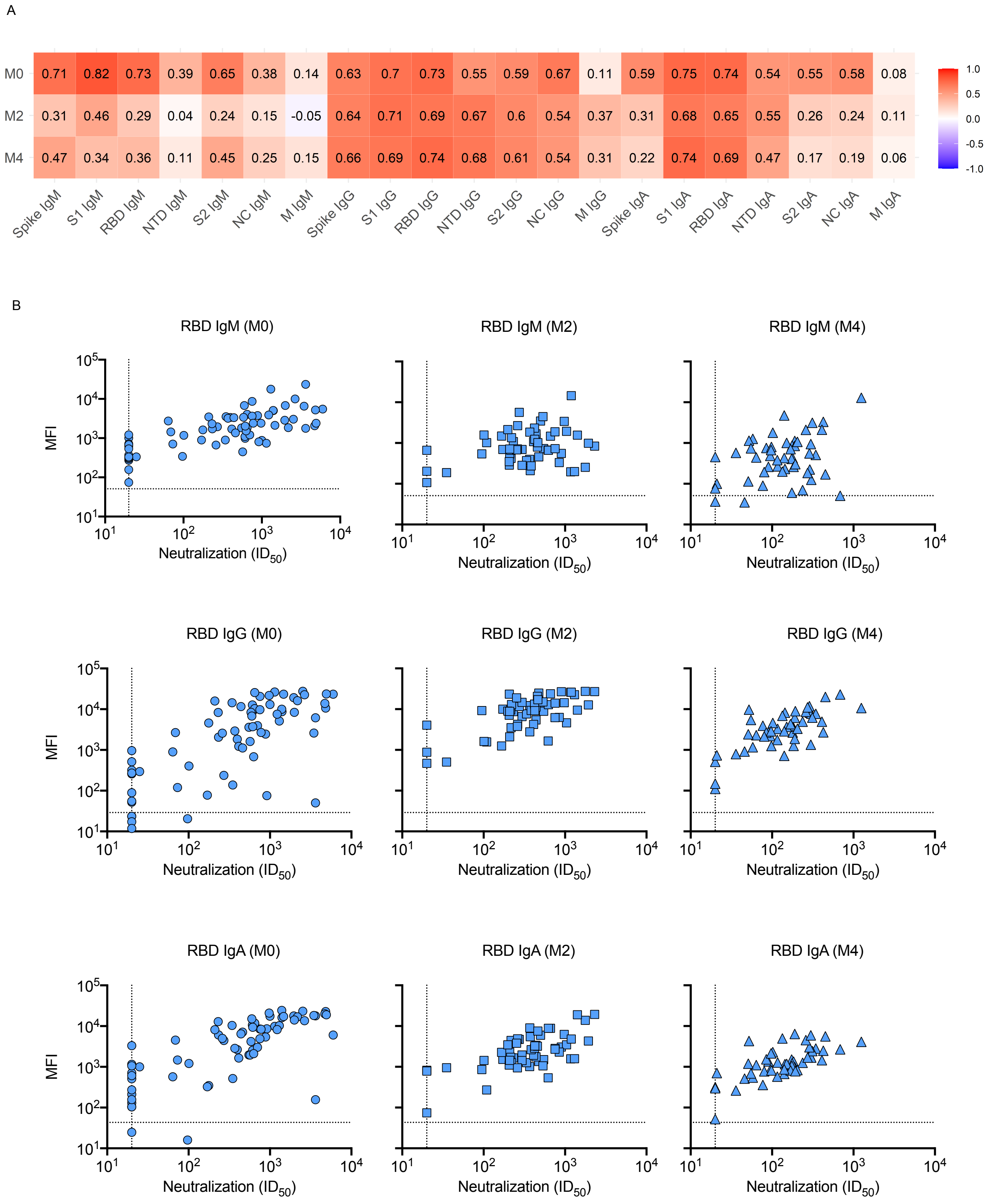

Supplemental Figure 3. Correlation between levels of SARS-CoV-2-specific antibodies and

neutralizing activity (ID_{50}) in sera from children and adolescents. Levels of IgM, IgG, and IgA antibodies to SARS-CoV-2 antigens were measured as mean fluorescence intensity using Luminex-based multiplex assays. Antibody-mediated neutralization activity was measured using a pseudovirus (614G) assay and is presented as 50% inhibitory dilution (ID_{50}). (a) Pearson's correlation coefficients (r) are shown between specific antibody measures and ID_{50}, each considered on the logarithmic scale. (b) Correlation plots between RBD-specific antibodies (IgM, IgG, IgA) and neutralization.

Supplemental Figure 4

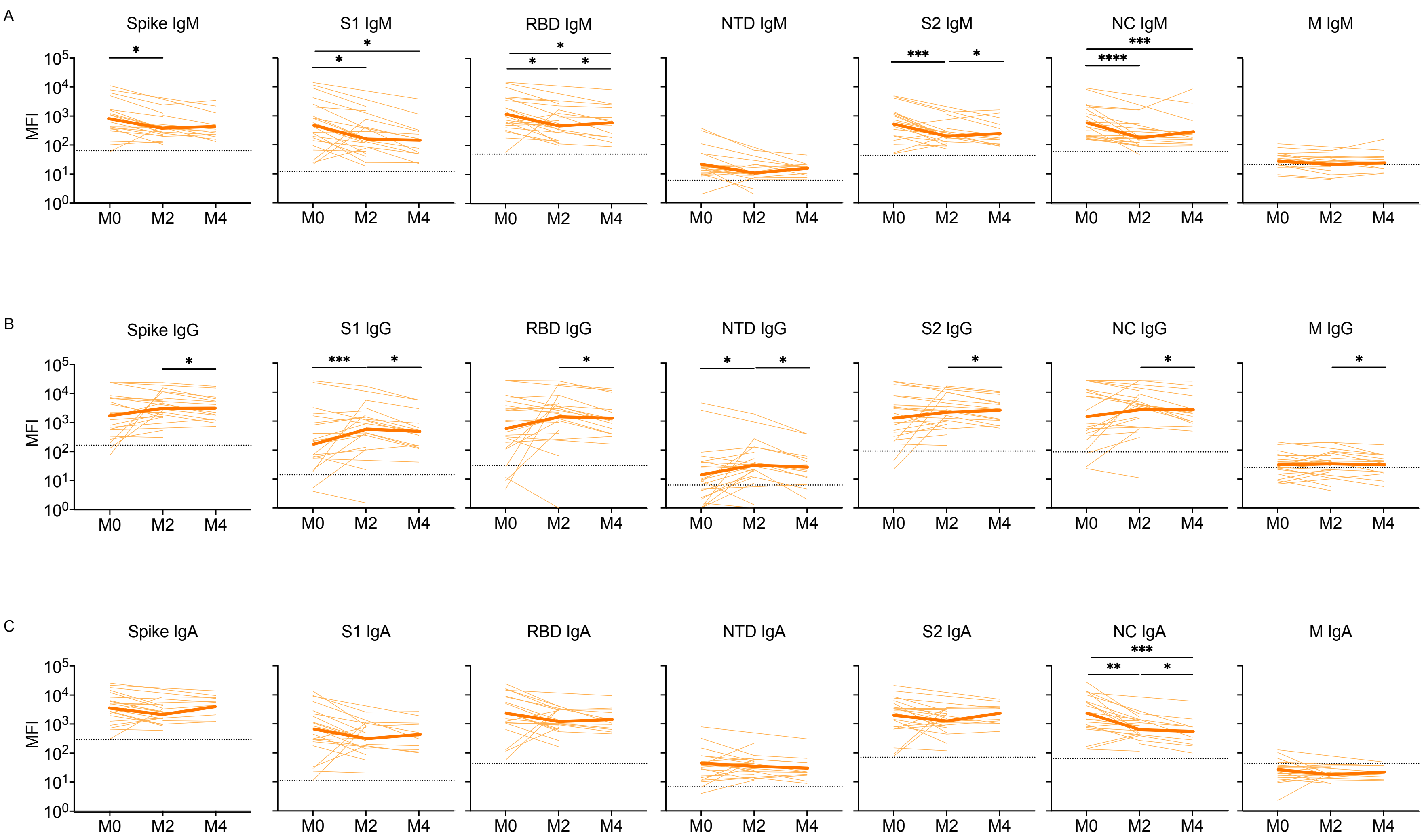

Supplemental Figure 4. SARS-CoV-2-specific binding antibodies in sera from adults. Specific binding to SARS-CoV-2 antigens (spike, subunit 1 (S1), subunit 2 (S2), receptor binding domain (RBD), N-terminal domain (NTD), subunit 2 (S2), nucleocapsid (NC), and membrane (M) proteins) was measured by a Luminex-based multiplex assay for IgM (**a**), IgG (**b**), and IgA (**c**) . Mean fluorescence intensity (MFI) of sera

from specific individuals (light orange lines) and the geometric mean in all individuals (thick orange lines) are shown at the time of acute infection (M0) and 2 months (M2) and 4 months (M4) after acute infection. The dotted lines indicate assay thresholds corresponding to the mean MFI plus three standard deviations in sera from 10 SARS-CoV-2-uninfected individuals. Levels of specific antibodies were compared across time points using Wilcoxon signed-ranks tests. * $p<0.05$; ** $p<0.01$; *** $p<0.005$; **** $p<0.0001$

Supplemental Figure 5

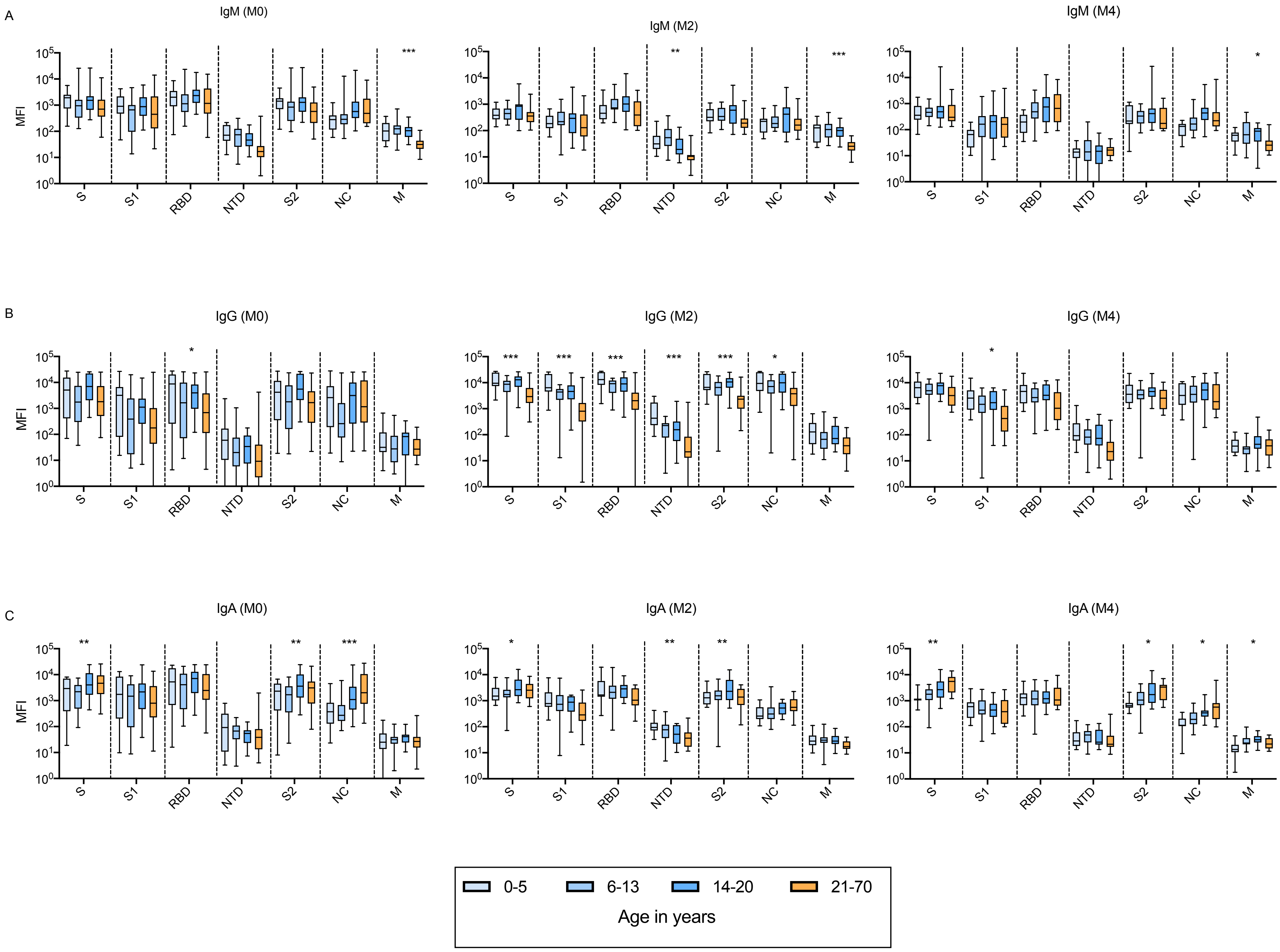

Supplemental Figure 5. SARS-CoV-2 binding antibodies in sera by age group. Specific binding to SARS-CoV-2 antigens (Spike (S), subunit 1 (S1), subunit 2 (S2), receptor binding domain (RBD), N-terminal domain (NTD), subunit 2 (S2), nucleocapsid (NC), and membrane (M) proteins) was measured by a Luminex-based multiplex assay for IgM (**a**), IgG (**b**), and IgA (**c**). The dotted lines indicate assay thresholds corresponding to the mean MFI plus three standard deviations in sera from 10 SARS-CoV-2-uninfected individuals. Levels of specific antibodies were compared across age category using ANOVA. * $p<0.05$; ** $p<0.01$; *** $p<0.005$; **** $p<0.0001$

Table S1. SARS-CoV-2-specific binding antibodies amongst children and adolescents (0-20 years of age)

		Acute Infection (M0; n=69)		Month 2 (M2: n=56)		Month 4 (M4; n=50)	
		Median (IQR) MFI		Median (IQR) MFI		Median (IQR) MFI	
IgM							
	Spike	1434.5	(658.8, 2047.3)	506.4	(266.3, 839.1)	472.2	(308.0, 783.3)
	Subunit 1	814.5	(331.8, 1609.5)	238.9	(147.9, 442.6)	127.5	(46.5, 265.3)
	RBD	1825.0	(803.5, 3398.3)	721.1	(414.3, 1504.1)	446.9	(194.8, 922.7)
	NTD	65.0	(28.3, 111.5)	32.4	(17.4, 65.1)	14.2	(6.9, 25.6)
	Subunit 2	1054.3	(566.5, 1682.3)	379.2	(225.7, 725.0)	358.1	(172.2, 624.2)
	NC	330.3	(193.3, 568.5)	222.8	(108.3, 359.5)	175.1	(97.0, 429.2)
	Membrane	105.0	(61.0, 164.0)	108.5	(54.8, 166.7)	66.0	(36.1, 114.6)
IgG							
	Spike	3995.0	(726.8, 12348.5)	9221.0	(4980.0, 15926.0)	6211.2	(3513.0, 9483.5)
	Subunit 1	876.3	(77.5, 3709.5)	5131.0	(2566.0, 7838.0)	1751.4	(836.6, 3375.6)
	RBD	2912.8	(294.8, 10687.0)	9360.0	(4520.0, 14450.5)	3494.3	(1941.4, 6721.1)
	NTD	41.5	(6.3, 92.5)	231.4	(113.7, 347.1)	77.5	(49.7, 217.0)
	Subunit 2	3120.0	(606.5, 11443.5)	8272.0	(4680.0, 13220.0)	3642.0	(2828.0, 6040.0)
	NC	962.8	(134.0, 4370.0)	8554.0	(4475.0, 15361.2)	3558.6	(1763.6, 7694.8)
	Membrane	30.8	(15.8, 107.5)	73.7	(38.2, 160.6)	31.8	(21.0, 57.3)
IgA							
	Spike	2834.5	(884.8, 6293.0)	1823.9	(1274.6, 3517.1)	1681.5	(1085.2, 3061.2)
	Subunit 1	1750.5	(347.5, 4579.0)	763.8	(528.1, 1517.7)	450.2	(266.4, 805.7)
	RBD	5006.0	(1060.0, 11851.0)	2267.0	(1403.0, 4331.0)	1204.0	(786.0, 2256.0)
	NTD	66.8	(23.3, 117.3)	81.3	(39.6, 118.5)	32.8	(22.5, 64.7)
	Subunit 2	2462.0	(548.0, 4450.0)	1510.9	(969.9, 2902.6)	1044.8	(613.4, 2108.7)
	NC	434.3	(216.3, 1109.8)	331.6	(231.7, 605.4)	245.1	(128.5, 360.0)
	Membrane	31.8	(19.0, 45.0)	28.8	(21.2, 41.6)	24.4	(14.9, 36.2)

IQR, interquartile range; MFI, mean fluorescence intensity; RBD, receptor binding domain; NTD, N-terminal domain; NC, nucleocapsid

Table S2. SARS-CoV-2-specific binding antibodies amongst adults (21-70 years of age)

		Acute Infection (M0; n=22)		Month 2 (M2: n=19)		Month 4 (M4; n=14)	
		Median (IQR) MFI		Median (IQR) MFI		Median (IQR) MFI	
IgM							
	Spike	702.1	(377.5, 1512.9)	357.3	(220.1, 479.1)	298.8	(229.5, 490.0)
	Subunit 1	448.5	(149.9, 1770.9)	128.5	(68.3, 351.8)	160.5	(50.0, 290.0)
	RBD	1167.2	(507.3, 4125.8)	389.0	(208.6, 1133.9)	654.5	(196.5, 2037.3)
	NTD	16.8	(10.6, 19.5)	10.5	(8.3, 11.7)	16.5	(10.5, 21.3)
	Subunit 2	572.8	(241.6, 1228.2)	191.0	(127.5, 283.0)	173.5	(107.3, 509.0)
	NC	486.6	(204.6, 1570.3)	162.3	(107.4, 238.2)	219.5	(151.0, 254.3)
	Membrane	31.4	(22.0, 42.9)	25.5	(17.8, 35.9)	25.5	(15.0, 36.5)
IgG							
	Spike	1815.8	(571.1, 7087.4)	2913.3	(1766.8, 5882.4)	3214.9	(1454.7, 6325.7)
	Subunit 1	176.8	(52.1, 2515.2)	802.8	(330.8, 1524.0)	424.3	(140.4, 775.1)
	RBD	687.8	(131.5, 3236.5)	2028.0	(965.2, 4022.3)	1042.5	(413.6, 2379.1)
	NTD	9.2	(2.7, 39.7)	22.0	(15.5, 67.6)	22.75	(11.6, 48.3)
	Subunit 2	1672.8	(324.8, 3565.9)	2322.5	(1019.4, 3104.2)	2549.2	(1069.6, 4262.7)
	NC	1165.0	(325.6, 11401.6)	3736.0	(1339.0, 6216.0)	1875.2	(953.7, 6292.2)
	Membrane	26.9	(16.6, 61.1)	37.8	(18.8, 71.7)	37.8	16.7, 62.8)
IgA							
	Spike	4678.0	(2053.0, 8322.0)	2530.0	(1360.0, 4053.0)	5531.0	(2357.0, 7912.0)
	Subunit 1	791.1	(251.6, 2699.8)	284.4	(177.2, 817.9)	381.5	(152.7, 1015.8)
	RBD	2459.3	(1083.0, 8974.4)	1043.2	(695.5, 2955.8)	1067.5	(681.1, 2804.4)
	NTD	38.4	(14.5, 72.3)	36.4	(18.1, 58.9)	21.5	(17.7, 45.3)
	Subunit 2	3096.2	(809.2, 4805.3)	1379.0	(762.6, 2628.9)	3334.0	(1166.0, 3834.0)
	NC	2009.5	(759.5, 9003.9)	554.5	(401.5, 1042.8)	569.3	(238.4, 801.2)
	Membrane	26.8	(16.3, 37.1)	17.3	(14.4, 24.9)	21.8	(16.1, 36.3)

IQR, interquartile range; MFI, mean fluorescence intensity; RBD, receptor binding domain; NTD, N-terminal domain; NC, nucleocapsid
